# Transcriptional Regulation of CYP3A4/2B6/2C9 Mediated via Nuclear Receptor PXR by Helicid and Its Metabolites

**DOI:** 10.1155/2015/797496

**Published:** 2015-04-22

**Authors:** Qun Chen, Hai-tang Xie, Yan Li, Guo Wang, Zhe Xu, Zhi-chen Pu, Hua Hu

**Affiliations:** ^1^Department of Clinical Pharmacy, Yijishan Hospital of Wannan Medical College, Anhui Provincial Center for Drug Clinical Evaluation, Wuhu, Anhui 241001, China; ^2^Traditional Chinese Medical Hospital, Wuhu, Anhui 241001, China; ^3^Department of Traditional Chinese Medicine, Yijishan Hospital of Wannan Medical College, Wuhu, Anhui 241001, China; ^4^Department of Clinical Pharmacology, Xiangya Hospital, Central South University, Changsha 410078, China; ^5^Institute of Clinical Pharmacology, Central South University, Changsha 410078, China

## Abstract

*Objective*. This study aims at establishing and validating an in vitro system to screen drug inducers of CYPs mediated via hPXR, as well as studying transcriptional regulation of CYPs mediated via hPXR by helicid and its two metabolites. *Methods*. Cloning the nuclear receptor hPXR and the promoters of CYP3A4, CYP2B6, CYP2C9, and inserting the trans-element to the upstream of firefly luciferase reporter gene of the pGL4.17 vectors, then cotransfecting the report vectors and hPXR expression plasmid to HepG2 cell line. After 24 hours, the transfected cells were treated with helicid (0.004, 0.04, and 0.4 *μ*mol/L) and its metabolite I and metabolite II (0.0004, 0.004, and 0.04 *μ*mol/L) for 48 h, while rifampin (10 *μ*mol/L) was included as the positive control and 0.1% DMSO as the negative control group. Cells were lysized and luciferase activity was determined using a dual luciferase reporter assay kit. *Results*. Helicid and its metabolites did not significantly increase promoter activities of CYP3A4, CYP2B6, and CYP2C9 in HepG2 cells transfected with PXR expression plasmid (*P* > 0.05). *Conclusion*. PXR-expressed CYP3A4, CYP2B6, and CYP2C9 dual luciferase reporter gene platforms were successfully established, and helicid and its metabolites I, II do not significantly induce the transcription of CYP3A4, CYP2B6, and CYP2C9.

## 1. Introduction

Biological organisms are continually exposed to potentially harmful chemicals called xenobiotics, including prescriptions and over-the-counter (OTC) drugs, herbal medicines, and environmental contaminants. Lipophilic xenobiotics are particularly dangerous, because they are able to accumulate to toxic concentrations over a period of time [1]. Cytochrome P450 (CYP450, CYP) are phase I metabolic enzymes that can catalyze oxidative and reductive metabolism of a variety of drugs, precarcinogen, and other xenobiotics, and the synthesis and metabolism of steroid hormones and arachidonic acid [2]. Through regulating the expression of liver CYP gene which achieve the detoxification and removal of xenobiotics, the expression products of these genes catalyze xenobiotics to polarity, prompting foreign chemicals easily excreted from the body. Members of the CYP superfamily often catalyze the first step in the detoxification of lipophilic xenobiotics [3] because of their broad substrate specificity and abundance in the liver, intestine, and tissues that are constantly exposed to environmental chemicals [4, 5]. Upregulation of CYP enzymes induced by xenobiotics is an integral component of the body's defense mechanism against chemical damage (chemoprotection) and enhances the detoxification of exogenous materials. In addition, CYP enzymes involve in the metabolism of multiple endogenous steroids bile acid and lipids.

The pregnane X receptor (PXR), which is a kind of ligand-activated transcription factors belonging to the nuclear receptor family, is an important part of the body's defense mechanism against toxic xenobiotics. In 1998, PXR was found in mice as a subfamily member of nuclear receptor family by Kliewer et al. [6]. The receptor was named as PXR because it was originally discovered that endogenous pregnane was its ligand. And it is also known as steroid and xenobiotic receptor (SXR) based on its activation by both steroid hormones and xenobiotic. While PXR is activated by ligand, the conformation changes and combines with 9-cis-retinoic acid receptor which is also known as retinoid X receptor (RXR), forms heterodimer to control gene transcription and trigger subsequent biological effects, and regulates genes of toxic substances that are involved in detoxification and excretion [7]. PXR is widely expressed in various important tissues and organs such as liver, intestine, kidney, lung, brain, pancreas, and placenta [8, 9].

Life and disease have very complex physiological and pathological processes, which involve multiple genes, multichannel process of molecular interaction networks function, to achieve the desired therapeutic effect being often more difficult to target through a single action. Thus, looking for modular drug targets will be an important development direction of drug research and development for multigene disorders associated “molecular group,” that is, network pharmacology [10]. Network pharmacology studies emphasize many effective drugs act via modulation of multiple proteins rather than single targets, highlighting overall thinking also shared by traditional Chinese medicine (TCM). Introducing network pharmacology in TCM will be easier to discover bioactive ingredients and endogenous/exogenous biomarkers, revealing molecular mechanisms and exploring scientific evidence of numerous herbs and herbal formulae in TCM on the basis of complex physiological and pathological processes [11–13].

CYP3A subenzymes are one of the most significant families involved in more than 50% of clinical drug metabolism. CYP3A gene can be induced by a lot of drugs, which is mediated by the PXR [14]. Many drugs can be used as ligand of PXR and activate the receptor PXR; these drugs directly combine with the ligand binding domain (LBD) forming heterodimers, then bind to the response element of the target gene promoter, upregulate the expression of transcription of target gene and the enzyme protein, and enhance the metabolic capacity of substrate. In addition to regulation of CYP3A gene, PXR also can regulate CYP2B and CYP2C genes, as well as the expression of phase II drug-metabolizing enzymes and drug transporters [15–17]. PXR receptor can mediate the formation of the enzyme CYP system; therefore, a drug may induce CYP enzymes by binding to the PXR receptor and activating it. The generated CYP enzymes will not only increase the metabolism of the drug itself, but also the coadministered drugs, which is called drug-drug interactions. As the metabolism of the drug accelerated, the clinical therapeutic effect is affected accordingly. Therefore, the PXR receptor can regulate the whole process of exogenous chemical reactions and drug metabolism.

**Figure 1:**
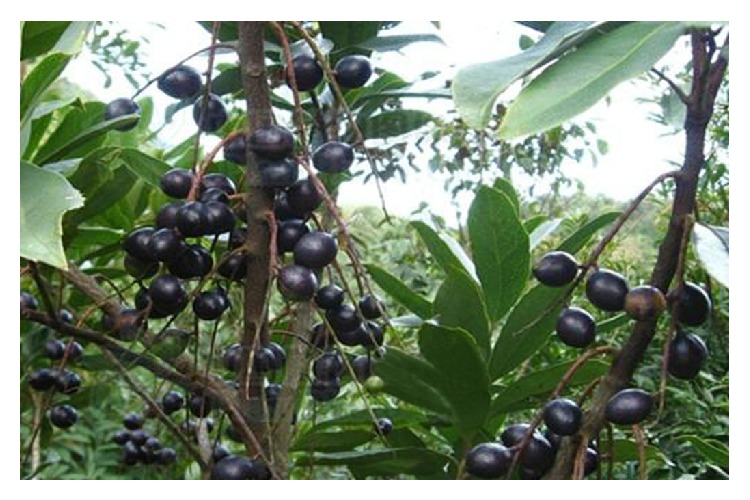
Fruits of Yunnan Helicia Nilagirica Beed in China.

**Figure 2:**
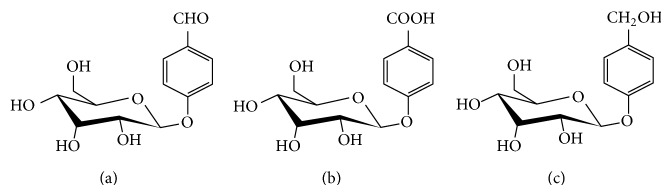
(a) is molecular structure of helicid, (b) is molecular structure of helicid oxidized metabolite (I), and (c) is molecular structure of helicid reduced metabolite (II).

The chemical name of helicid is 4-formylphenyl-*β*-D-allopyranoside, a kind of aromatic phenolic glycosides. It is an active ingredient extracted from the fruits from Yunnan Helicia Nilagirica Beed (synonyms Helicia Erratica Hook) in China (Figure 1). Thousands of years ago, the Jingpo people in Yunnan had already used it to reduce swelling and alleviate pain. The chemical structure of helicid is a kind of monomer glucoside (Figure 2(a)). Pharmacological and clinical research found that helicid can restore the imbalance between the cerebral cortex excitability and inhibition and people use it to alleviate pain and induce sleep or sedation [18–20]. Helicid tablets belong to the state OTC drugs, widely used for the adjuvant treatment of insomnia, neurasthenia, migraine, headache, and other symptoms; now as a novel treatment for depression and neuropathic pain, a series of research has been carried out to study the related pharmacological effects and mechanism on helicid and its derivatives. Studies showed that helicid has 2 kinds of metabolites in the human body: oxidized metabolites (Figure 2(b)) and the reduced metabolites (Figure 2(c)).

In the view of many drugs there exists induction or inhibition on hepatic drug metabolizing enzyme (especially CYP); this leads to serious drug-drug interactions (DDI) in clinical practice, especially during postmarketing stage. Sometimes abnormal plasma concentration and poisoning phenomenon even appeared. The Food and Drug Administration (FDA) of USA and China Food and Drug Administration (CFDA) have taken related measures, such that the drug's ability in inducing or inhibiting hepatic drug metabolizing enzyme must be studied in the process of new drug development in order to ensure the safety of the drug. Since the use of natural medicines has become more and more widely accepted, full attention should be paid to drug-induced adverse interactions.

At present, researches of helicid and its metabolites are mainly focused on clinical pharmacodynamics and less researches on the DDI. This paper was based on the molecular mechanisms in which drugs induced CYP450 enzymes, constructed luciferase reporter gene vectors containing CYP3A4, CYP2B6, and CYP2C9 gene promoter regulatory sequence with molecular and cell biology methods, established screening system in vitro of hPXR-mediated CYP3A4, CYP2B6, and CYP2C9 drugs inducers, and further used it to study helicid and metabolites on hPXR-mediated CYP3A4, CYP2B6, and CYP2C9 induced effects, to predict whether helicid can cause DDI in the human body or not.

## 2. Materials and Methods

### 2.1. Chemicals

HepG2 cells were purchased from the Type Culture Collection of the Chinese Academy of Sciences, Shanghai, China. DMEM high glucose medium, fetal bovine serum, and trypsin were purchased from Hyclone. TIANgel Midi Purification Kit and T4DNA ligase were purchased from TIANGEN Biotech (Beijing) Co., Ltd. Restriction endonuclease, PrimeSTAR GXL DNA polymerase, and Taq DNA polymerase were purchased from TakaRa Biotechnology (Dalian) Co., Ltd. Plasmid Mini Kit and EndoFree Plasmid Kit were purchased from Omega. Lipofectamine 2000 reagent was purchased from Invitrogen Trading (shanghai) Co., Ltd. Reverse Transcription System, Dual-Luciferase Reporter Assay System, and pGEM-T Vector System were purchased from Promega. Rifampicin was purchased from Sigma (America). Helicid (H) and its metabolites (H-I, H-II) were provided by Kunming Baker-Norton Pharmaceutical Co., Ltd.

### 2.2. Expression Vectors Construction

Human liver tissue was obtained from The Third Xiangya Hospital of Central South University. Total RNA was isolated from the human liver tissue. We applied hPXR-R specific downstream primer for reverse transcription (Promega). The hPXR PCR reactions were performed as follows: 10 *μ*L 5×PrimeSTAR GXL Buffer, 200 *μ*M each dNTP, $\begin{array}{c} 25\;\text{ng}\sim 750\;\text{ng} \end{array}$ cDNA, 10 pmol of each primer (hPXR, Table 1), and 1.25 U PrimeSTAR GXL DNA polymerase, in a total volume of 50 *μ*L. The mixture was subjected to 35 cycles consisting of a 10 sec denaturing step at 98°C, a 15 sec annealing step at 60°C, and a 90 sec elongation step at 68°C in a thermal cycler. Amplification products were resolved by agarose gel (1%) electrophoresis and detected by ethidium bromide. The target band was visualized under UV light and photographed with a computer-assisted camera, cut the target band from the agarose gel (about 1.3 kb), and then completed purification and recovery of the target DNA band. Plussed A at the 3′ end of the PCR product ligated the pGEM-T vector and transformed competent JM109 using standard protocol. After 12–16 hours, we picked the monoclone into the Amp^+^-LB liquid culture medium and shaked the culture for 12 hours. After colony PCR and restriction enzyme digestion, it was sent for sequencing. Double digested the cloned plasmid DNA which had been verified by sequencing and pcDNA3.1−myc/his B (−) (Invitrogen) with BamH I and EcoR I. After electrophoretic separation, cut the target bands from the agarose gels, respectively, then purify, and recover the two. We ligated the digested target DNA and pcDNA3.1−myc/his B (−), then transformed into* E. coli*. Plasmid DNA was extracted; electrophoresis detection was done after double digestion with BamH I and EcoR I. A large number of plasmid DNA were extracted while it had been verified by sequencing. The expression vector was named hPXRΔATG-pcDNA3.1B (−).

### 2.3. Luciferase Reporter Gene Vector Construction

Primers were designed according to the literature (sequences showed in Table 1) [21–23]. Human gDNA as a template and 3A4-1-F/3A4-1-R and 3A4-2-F/3A4-2-R as primer pairs amplified, respectively, distal promoter regulatory sequences (−7836 ~ −7208 bp) and proximal promoter regulatory sequences (−362 ~ +53 bp) of CYP3A4. Ligate them one after another in the Nhe I/Bgl II and Bgl II/Hind III sites of pGL4.17 vector. 2B6-2-F/2B6-2-R and 2B6-1-F/2B6-1-R as primer pairs amplified, respectively, proximal promoter regulatory sequences (−2318 ~ −2155 bp) and distal promoter regulatory sequences (−8.5 ~ −8.6 kb) of CYP2B6. Ligate them one after another in the Kpn I/Xho I and Bgl II/Hind III sites of pGL4.17 vector. 2C9-1-F/2C9-1-R and 2C9-2-F/2C9-2-R as primer pairs amplified, respectively, distal promoter regulatory sequences (−2899 ~ −2883 bp) and proximal promoter regulatory sequences (−1839 ~ −1824 bp) of CYP2C9. Ligate them one after another in the Hind III and Kpn I/Hind III sites of pGL4.17 vector. Vectors were named CYP3A4-promoter-Luc, CYP2B6-promoter-Luc, and CYP2C9-promoter-Luc. Verified by restriction analysis and sequencing, a large number of plasmids were extracted.

### 2.4. Cell Culture and Transient Transfections

The human hepatocellular carcinoma cell line HepG2 cells were obtained from the Type Culture Collection of the Chinese Academy of Sciences, Shanghai, China. HepG2 cells were maintained at 37°C, 5% CO_2_ in high glucose Dulbecco's modified Eagle's medium (DMEM) with glutamine (Hyclone), supplemented with 10% fetal bovine serum. For transient transfection, 24 hours before transfection, HepG2 cells were seeded in 24-well plates at 0.5–2 × 10^5^ cells per well. Transfections were performed in triplicate using 400 ng CYP2B6/CYP2C9/CYP3A4-promoter-Luc, 100 ng hPXRΔATG-pcDNA3.1 B (−), 20 ng internal control PRL-TK, and 2 *μ*L lipofectamine 2000 in a well. Four to six hours after transfection, the cells were washed in DMEM and DMEM + 10% FBS medium was replaced and treated with drugs 20 hours later.

### 2.5. Test Drug Treatment and Detecting Luciferase Activity

Rifampicin, helicid, and its metabolites were prepared with DMSO, respectively. At 24 h posttransfection, discarding the original medium, DMEM + 10% FBS medium with 0.004, 0.04, and 0.4 *μ*mol/L helicid(H)/0.0004, 0.004, and 0.04 *μ*mol/L metabolites (H-I, H-II) was replaced, and drugs were incubated with the cells for 48 h. DMSO concentration in each drug treated group was controlled to 0.1%, meantime, we set containing 0.1% DMSO as the vehicle control group and containing 10 *μ*mol/L rifampicin-0.1% DMSO as the positive group. The cells were harvested at 48 h after the transfection. The luciferase activity in the cleared cell lysate was measured with the dual luciferase assay kit (Promega), and firefly luciferase and Renilla luciferase activity values were determined. Cell transfection and luciferase activity assay was repeated three times in triplicate.

### 2.6. Statistical Analysis

The efficiency of transfection in each of the treated cells was corrected by the ratio of firefly luciferase activity to Renilla luciferase activity. Fold activation was defined as the ratio of sample activity value of the corrected transfection efficiency of each group to the same sample activity value of the corrected transfection efficiency of 0.1% DMSO vehicle control group. All results are presented as the mean ± standard deviation (SD). Data were analyzed using ANOVA analysis of variance, with a value of *P* < 0.05 considered significant by SPSS 18.0, pairwise comparisons using LSD method. Each group was compared with the control group whether there is a statistically significant difference.

## 3. Results

### 3.1. hPXRΔATG-pcDNA3.1B (−) Expression Vector Construction

**Figure 3:**
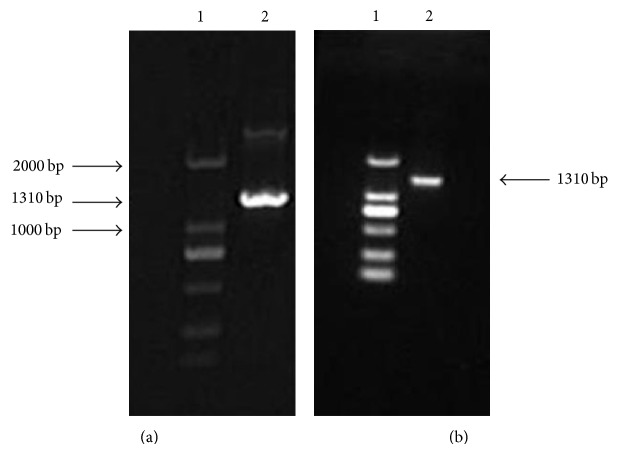
PCR amplification hPXR: (a) hPXR band before recovery (1310 bp); (b) hPXR band after recovery (1 is DNA Marker DL2,000 and 2 is hPXR band).

**Figure 4:**
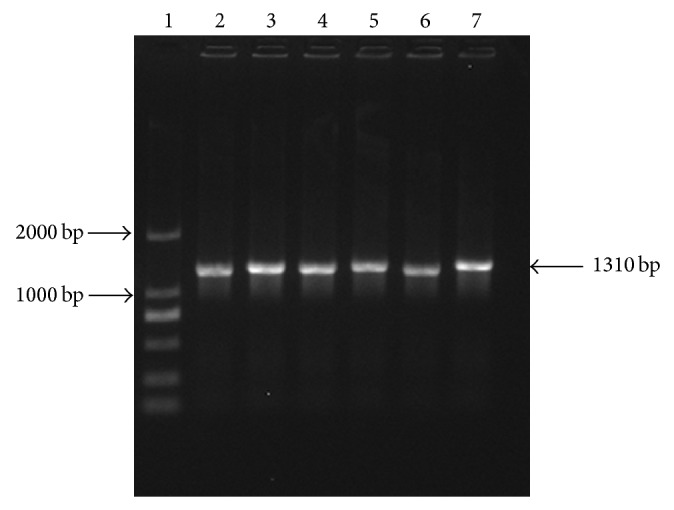
Colony PCR of hPXRΔATG-pcDNA3.1B (−) (1 is DNA Marker DL2,000 and 2−7 are hPXR bands).

**Figure 5:**
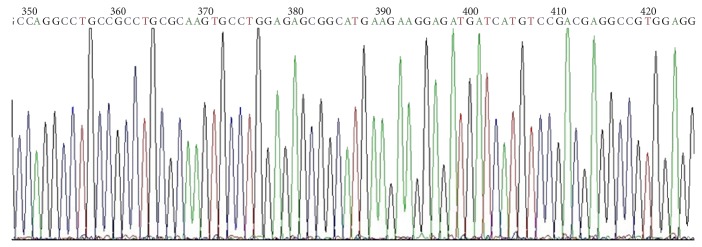
The sequencing results of hPXRΔATG-pcDNA3.1B (−).

Total RNA was extracted from human liver tissue and amplified PXR target fragment (1.3 kb) by RT-PCR. Amplification product was analysed by agarose gel (1%) electrophoresis; nonspecific bands disappeared after recovery (Figure 3). PCR product ligated the pGEM-T vector and double digested the cloned plasmid DNA which had been verified by sequencing and pcDNA3.1−myc/his B (−) with BamH I and EcoR I. We ligated the digested target DNA and pcDNA3.1−myc/his B (−), then transformed into* E. coli*. After colony PCR and verification by sequencing, the expression vector hPXRΔATG-pcDNA3.1B (−) is successfully constructed (Figures 4 and 5).

### 3.2. Dual Luciferase Reporter Gene Vector Construction

**Figure 6:**
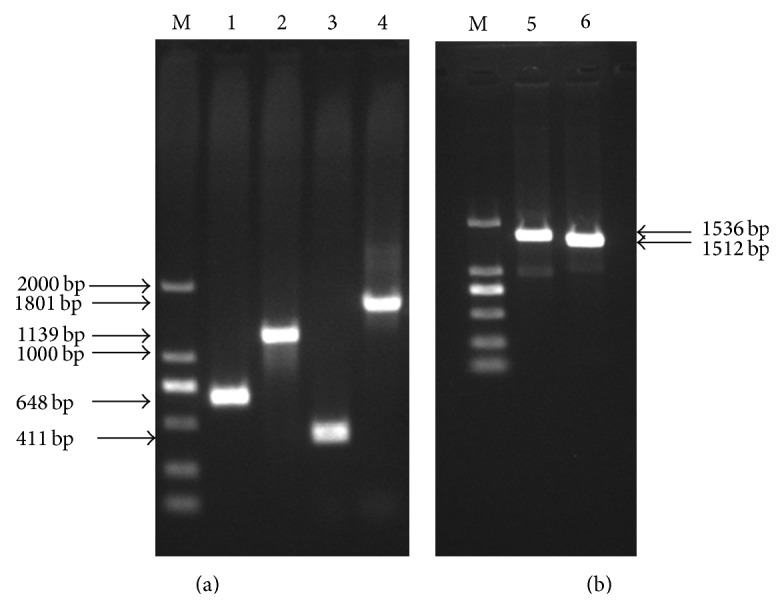
PCR amplified CYP3A4, CYP2B6, and CYP2C9 promoter proximal and distal regulatory sequences (M is DNA Marker DL2,000, 1, 2 are CYP3A4 promoter proximal and distal bands, 3, 4 are CYP2B6 promoter proximal and distal bands, and 5, 6 are CYP2C9 promoter proximal and distal bands).

**Figure 7:**
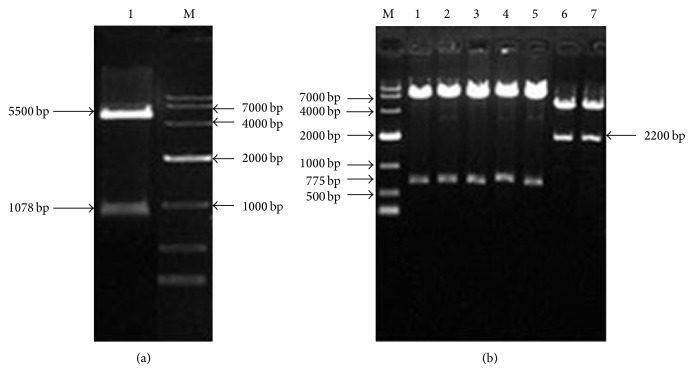
Verification of restriction analysis of CYP3A4-promoter-Luc, CYP2B6-promoter-Luc, and CYP2C9-promoter-Luc: (a) is enzyme digestion analyses with Kpn I and Hind III of CYP3A4-promoter-Luc (M is DNA Marker DL10,000 and 1 is 1078 bp DNA band of CYP3A4); (b) is enzyme digestion analyses of CYP2B6-promoter-Luc and CYP2C9-promoter-Luc (M is DNA Marker DL10,000, 1, 2, 3, 4, 5 are 775 bp DNA bands of CYP2C9, and 6, 7 are 2200 bp DNA bands of CYP2B6).

Human gDNA as a template amplified, respectively, distal promoter regulatory sequences and proximal promoter regulatory sequences of CYP3A4, CYP2B6, and CYP2C9.1% agarose gel electrophoresis analysis showed suitable size DNA bands (Figure 6). Insert distal and proximal regulatory sequences into the linearized pGL4.17 vector. CYP3A4 and CYP2B6 were verified by double restriction enzyme digestion analyses with Kpn I and Hind III; CYP2C9 was verified by single restriction enzyme digestion analyses with EcoR V (Figure 7). After verification by sequencing, promoter sequences connected with the vector PGL4.17 successfully; CYP3A4-promoter-Luc, CYP2B6-promoter-Luc, and CYP2C9-promoter-Luc vectors are successfully constructed.

### 3.3. Induced Effects of CYP3A4/2B6/2C9 Mediated via PXR by Different Concentrations of H, H-I, and H-II

**Figure 8:**
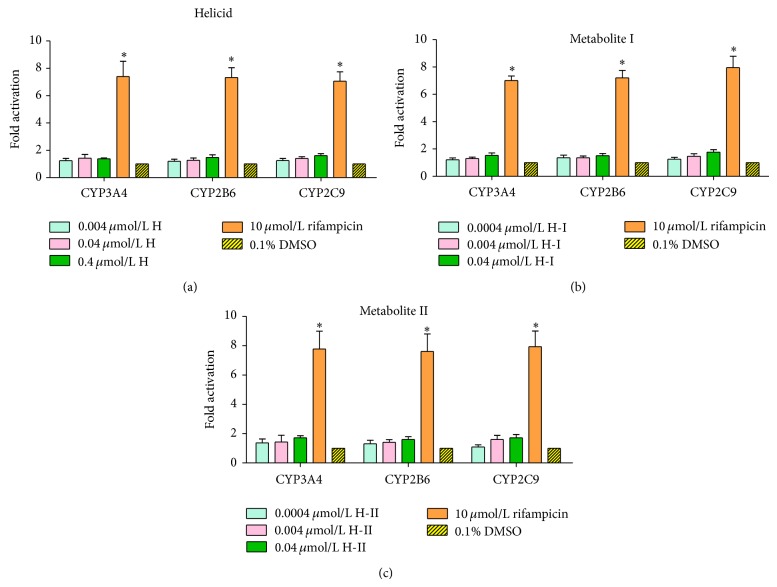
Induced effects of CYP3A4/2B6/2C9 mediated via PXR by helicid (a) and its metabolites I (b), II (c) ($\overset{-}{X}\pm S$). There was a significant difference between positive control and 0.1% DMSO treatment group (^*^ *P* < 0.01), but there was no significant difference between helicid and its metabolites I, II and 0.1% DMSO treatment group (*P* > 0.05).

Twenty-four hours following transfection, cells were treated with 0.004, 0.04, and 0.4 *μ*mol/L helicid(H)/0.0004, 0.004, and 0.04 *μ*mol/L metabolites (H-I, H-II) for 48 h, detected dual luciferase activity. The results of specific activity values of various concentrations groups are shown in Figure 8. At the three concentrations, no significant difference was found between helicid and its metabolites I, II and 0.1% DMSO vehicle control group (*P* > 0.05); the same result was shown between the three concentrations (*P* > 0.05). 10 *μ*mol/L rifampicin induced CYP3A/CYP2B6/CYP2C9 expression increased 7-8 times. There was statistically significant difference between 10 *μ*mol/L rifampicin positive control group and various concentrations groups or 0.1% DMSO vehicle control group (*P* < 0.001). Helicid and its metabolites I, II cannot significantly induce the transcription of CYP3A4, CYP2B6, and CYP2C9.

## 4. Discussion

There are almost more than 90% of clinical drugs metabolized through CYP3A4, CYP2C9, or CYP2B6. The inducers or inhibitors of hepatic drug metabolizing enzymes can speed up or slow down the metabolism of the substrates or other drugs. These constitute the material basis of the drug metabolism in the body and interactions between the drugs [24]. Therefore, the impact of drugs on the activity of CYP enzymes is one of the main reasons leading to clinical DDI. CYP450 enzymes are composed of the enzyme protein encoded by the gene superfamily and involved in the biotransformation of almost all endogenous and exogenous substances. CYP gene can be induced by a lot of drugs; studies have shown that this induction is mediated via the nuclear receptor PXR [14]. Many clinical drugs can be ligand of PXR to activate the receptor, to regulate the expression of CYP gene, such as antibiotic, psychotropic drugs, and antidiabetic drugs. Metabolism's study of traditional Chinese medicine is becoming one of the hot areas of the traditional Chinese medicine research in recent years; more and more reports appeared. For example, ginkgo, coumarin, and* Hypericum perforatum* can inhibit the expression of multiple isoforms of CYP450, and glycyrrhizic acid and angelica polysaccharide can induce some of them [25].

Helicid used as a kind of traditional medicine has a long history, and it is a chemical monomer with potential medicinal value whose activity is similar to hyperoside and has the basis of long-term clinical applications and new use patent. Helicid used for the treatment of depression was authorized by the State Patent Office in January 2005 [26]. Therefore, there is a very important clinical significance for predicting DDI through the research of helicid act on CYP enzymes.

This paper was based on the molecular mechanisms in which drugs induced CYP450 enzymes and constructed luciferase reporter gene vectors containing CYP3A4, CYP2B6, and CYP2C9 gene promoter regulatory sequence with molecular and cell biology methods. Reporter gene vectors were cotransfected with PXR expression vector into HepG2 cells in vitro, were treated with hPXR agonist rifampicin, and regulated CYP3A4, CYP2B6, and CYP2C9 promoter regulatory sequences by activating the exogenous expression vector hPXR to significantly increase the expression of luciferase. Nuclear receptor PXR-expressed CYP3A4, CYP2B6, and CYP2C9 dual luciferase reporter gene platforms were successfully established and used the reporter gene platforms to detect helicid and its metabolites I and II. At the three concentrations, no significant difference was found between helicid or its metabolites I, II and 0.1% DMSO vehicle control group (*P* > 0.05); it is shown that helicid and its metabolites I, II cannot significantly induce the transcription of CYP3A4, CYP2B6, and CYP2C9 and prompt the probability that clinical DDI of helicid and its metabolites induce CYP enzymes mediated by hPXR which is small. This study laid the theoretical foundation for predictions of the development and DDI of helicid. This study successfully established a cell platform which can screen CYP3A4/CYP2C9/CYP2B6 inducers and used it to verify helicid. The results show that the effect of the positive drug is obvious; helicid displays a negative result. Combination drugs of helicid are proven to be safe. The screening system in vitro provides useful information for clinical drugs, especially the development of new drugs and other therapeutic agents.

**Figure 9:**
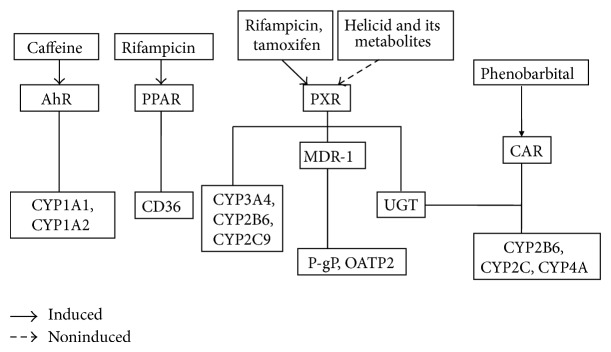
Network diagram of regulation of gene expression mediated via nuclear receptors by inducers.

Discover AhR, PXR, and CAR which are closely relevant to drug-metabolizing enzymes and transcription activation [27, 28] (Figure 9). AhR is the ligand-activated transcription factor of bHLH PAS family; CAR and PXR are members of nuclear hormone receptor superfamily. The two receptors CAR and PXR are closely related to each other in the regulation of drug-metabolizing enzymes and have overlapping functions [29, 30]. Mueller et al. [31] found that the induction of CYP2b10, CYP3a11, CDC20, and Cdk1 depend on both CAR and PXR, but AACS enzyme is only inducted by the PXR. The PXR also regulates the expression of drug metabolizing enzymes which include UDP-glucuronosyltransferase enzyme (UGT), sulfotransferase (SULT), and glutathione-S-transferase (GST) [32] and transporter proteins like P-glycoprotein (P-gP) [33] and organic anion transporter 2 (OATP2) [34]. This paper focuses on hPXR-mediated drug metabolizing enzymes CYP3A4, CYP2B6, and CYP2C9 inducing activity screening, studies are lacking in other areas, and follow-up will continue to be studied.

## 5. Conclusion

A convenient, economical, and new high-throughput in vitro screening system is established and validated to investigate the CYP enzyme-inducing activity and was successfully applied to investigate helicid and its metabolites. The screening system is a useful tool for the prediction of potential drug-drug interactions of clinical therapeutic agents.

## Figures and Tables

**Table 1:** Primer table of vectors construction.

Primers name	Sequence (5′→3′)
hPXR-F	CTG**GAATTC**GCCACCATGGAGGTGAGACCCAAAGAAAGC
hPXR-R	CTG**GGATCC**ACGCTACCTGTGATGCCGAACAAC
3A4-1-F	TAT**GCTAGC**GCTGGTTGCTGGTTTATTC
3A4-1-R	TAT**AGATCT**TCTCGTCAACAGGTTAAAGG
3A4-2-F	ATTGCTGGCTGAGGTGGTTG
3A4-2-R	ATAT**AAGCTT**CTGTGTTGCTCTTTGCTG
2B6-1-F	CTG**GGTACC**CTTTCTCCATCCACAAAATGG
2B6-1-R	CTG**CTCGAG**GATGCTGATTCAGGGAATGGA
2B6-2-F	CTG**AGATCT**CTGCAATGAGCACCCAATCTTA
2B6-2-R	CTG**AAGCTT**CTGCACCCTGCTGCAGCCT
2C9-1-F	TG**GGTACC**CCATAGAATGTACAACACAAAG
2C9-1-R	CTAGTGAGGTTATTTCCATTTCT
2C9-2-F	CAAAAGGGACATGAGGTGT
2C9-2-R	CTTG**AAGCTT**TCTCTTCTTGTTAAGACAACCA

^*^Boldfaces for the restriction sites.
